# Disparities in the non-laboratory INTERHEART risk score and its components in selected countries of Europe and sub-Saharan Africa: analysis from the SPICES multi-country project

**DOI:** 10.1093/ehjopen/oead131

**Published:** 2023-12-05

**Authors:** Hamid Y Hassen, Steven Abrams, Geofrey Musinguzi, Imogen Rogers, Alfred Dusabimana, Peter M Mphekgwana, Hilde Bastiaens, Scaling-up Packages of Interventions for Cardiovascular diseases in Europe and Sub-Saharan Africa (SPICES) study investigators, Hilde Bastiaens, Hamid Y Hassen, Naomi Aerts, Sibyl Anthierens, Kathleen Van Royen, Caroline Masquillier, Jean Yves Le Reste, Delphine Le Goff, Gabriel Perraud, Harm van Marwijk, Elisabeth Ford, Tom Grice-Jackson, Imogen Rogers, Papreen Nahar, Linda Gibson, Mark Bowyer, Almighty Nkengateh, Geofrey Musinguzi, Rawlance Ndejjo, Fred Nuwaha, Tholene Sodi, Peter M Mphekgwana, Nancy Malema, Nancy Kgatla, Tebogo M Mothiba

**Affiliations:** Department of Family Medicine and Population Health, Faculty of Medicine and Health Sciences, University of Antwerp, Doornstraat 331, Wilrijk 2610, Belgium; Department of Family Medicine and Population Health, Faculty of Medicine and Health Sciences, University of Antwerp, Doornstraat 331, Wilrijk 2610, Belgium; Interuniversity Institute for Biostatistics and Statistical Bioinformatics, Data Science Institute, Hasselt University, Diepenbeek 3590, Belgium; Department of Family Medicine and Population Health, Faculty of Medicine and Health Sciences, University of Antwerp, Doornstraat 331, Wilrijk 2610, Belgium; Department of Disease Control and Environmental Health, School of Public Health, Makerere University, Kampala, Uganda; Department of Primary Care and Public Health, Brighton and Sussex Medical School, Brighton, UK; Department of Family Medicine and Population Health, Faculty of Medicine and Health Sciences, University of Antwerp, Doornstraat 331, Wilrijk 2610, Belgium; Research Administration and Development, University of Limpopo, Polokwane 0700, South Africa; Department of Family Medicine and Population Health, Faculty of Medicine and Health Sciences, University of Antwerp, Doornstraat 331, Wilrijk 2610, Belgium

**Keywords:** Cardiovascular disease, Risk score, INTERHEART, Disparities, Europe, Sub-Saharan Africa

## Abstract

**Aims:**

Accurate prediction of a person’s risk of cardiovascular disease (CVD) is vital to initiate appropriate intervention. The non-laboratory INTERHEART risk score (NL-IHRS) is among the tools to estimate future risk of CVD. However, measurement disparities of the tool across contexts are not well documented. Thus, we investigated variation in NL-IHRS and components in selected sub-Saharan African and European countries.

**Methods and results:**

We used data from a multi-country study involving 9309 participants, i.e. 4941 in Europe, 3371 in South Africa, and 997 in Uganda. Disparities in total NL-IHRS score, specific subcomponents, subcategories, and their contribution to the total score were investigated. The variation in the adjusted total and component scores was compared across contexts using analysis of variance. The adjusted mean NL-IHRS was higher in South Africa (10.2) and Europe (10.0) compared to Uganda (8.2), and the difference was statistically significant (*P* < 0.001). The prevalence and per cent contribution of diabetes mellitus and high blood pressure were lowest in Uganda. Score contribution of non-modifiable factors was lower in Uganda and South Africa, entailing 11.5% and 8.0% of the total score, respectively. Contribution of behavioural factors to the total score was highest in both sub-Saharan African countries. In particular, adjusted scores related to unhealthy dietary patterns were highest in South Africa (3.21) compared to Uganda (1.66) and Europe (1.09). Whereas, contribution of metabolic factors was highest in Europe (30.6%) compared with Uganda (20.8%) and South Africa (22.6%).

**Conclusion:**

The total risk score, subcomponents, categories, and their contribution to total score greatly vary across contexts, which could be due to disparities in risk burden and/or self-reporting bias in resource-limited settings. Therefore, primary preventive initiatives should identify risk factor burden across contexts and intervention activities need to be customized accordingly. Furthermore, contextualizing the risk assessment tool and evaluating its usefulness in different settings are recommended.

## Graphical Abstract

**Graphical Abstract:**
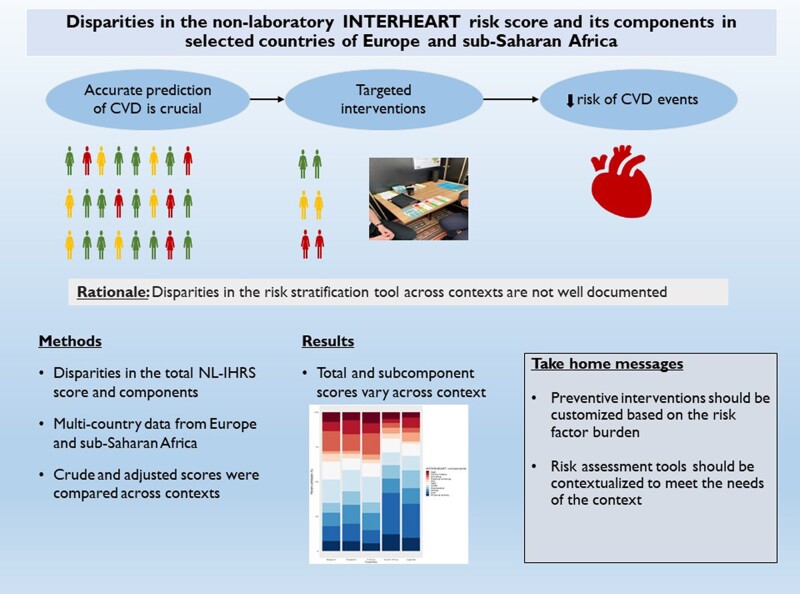

## Introduction

Despite improvements in healthcare systems, cardiovascular diseases (CVDs) continue to account for a large share of the global disease burden. In 2019, ∼523 million CVD cases, 18.6 million deaths, and 393 million disability adjusted life years were estimated, making CVDs the leading cause of adult morbidity and mortality.^1^ Among all CVD deaths, preventable causes including ischaemic heart disease and stroke are most prevalent.^1,2^

The burden of CVDs greatly varies across regions, countries, and time periods. Between 1990 and 2019, the age standardized CVD deaths declined in high-income and some middle-income countries. However, the trend is different in most low- and middle-income countries (LMICs), where a rise in the burden contributes to three-fourths of all global CVD deaths.^2,3^ There is an enormous discrepancy in CVD incidence, progression, and mortality in low-, middle-, and high-income countries.^4,5^ Although the burden of risk factors is lowest, the CVD-event rate and mortality are considerably higher in low-income countries (LICs).^4^ Better control of lifestyle risks, early detection, and frequent use of efficacious pharmacologic treatment for metabolic risk factors could mitigate the higher risk factor burden in high-income countries (HICs).

The variation in CVD burden observed between countries and regions could be due to socioeconomic variations, epidemiological and demographic transitions, and lifestyle differences because of urbanization and globalization.^2,3,6^ Socioeconomic status is associated with differences in risk factor burden, CVD incidence, and outcomes, including mortality.^7,8^ Although people with a lower level of education in LMICs have better overall risk factor profiles, they have higher incidence and mortality from CVD because of limited access to quality healthcare.^7^ The better risk factor profile in LMICs might be due to lack of regular medical check-up leading to a low detection rate. Attempts to tackle the global burden of CVD must emphasize effective control in high-risk groups.^9^ Detection and treatment of modifiable risks at an earlier stage could help to halt the early onset of CVD and complications associated with it.^10^

Accurate measurement of CVD risk is vital in order to plan appropriate lifestyle interventions and treatment of modifiable risks.^11^ Determining the risk at an individual level helps to better arrange further laboratory and imaging investigations and management with the greatest accuracy. Furthermore, at population level, it facilitates the decision making process in public health planning, development, and implementation of targeted interventions.^12^ To improve early detection and reach vast majorities, a simple tool is needed that can be utilized by non-healthcare professionals. McGorrian *et al*.^13^ developed a tool called the INTERHEART risk score (IHRS) to predict the risk of CVD among apparently healthy adults. The non-laboratory based tool (NL-IHRS) has nine components related to a person’s age, family history of CVD, lifestyle including smoking, dietary and leisure time activity, psychosocial factors, metabolic risks including history of diabetes mellitus (DM), high blood pressure (HBP), and physical measures (waist-to-hip ratio; WHR).

Using the NL-IHRS, Yusuf *et al*.^4^ indicated that the overall risk score is highest in HICs, intermediate in middle-income countries (MICs), and lowest in LICs, whereas the incidence of major CVD events was found to be in the opposite direction. However, studies that compare the variation in the overall risk score as well as specific components (behavioural, metabolic, and etc.) across contexts are scant. The self-reported nature of the majority of these components (DM, HBP, depression, and etc.) might impact the total score particularly in LICs as apparently healthy people usually do not have regular medical check-ups.^14^ The authors who developed the NL-IHRS also acknowledged the limitation that historical recall of HBP and DM may be considered inferior to direct measurement.^13^ They justified the approximation of the prevalence of self-reported and objectively measured hypertension and diabetes referring to a few studies.^15–17^ However, all those studies were conducted in HICs, which does not reflect the context of LMICs. Thus, it is imperative to assess the discrepancies in the NL-IHRS component risk scores across contexts to improve the usefulness of the tool. Furthermore, identifying the variation in component risk scores could help to determine the burden across contexts and to develop and implement targeted preventive interventions. Therefore, this study investigated variation in overall CVD risk, measured through the NL-IHRS, and its specific component scores in selected sub-Saharan African countries (Uganda and South Africa) and vulnerable communities in high-income European countries (Belgium, England, and France). Furthermore, we described the variation in study approach, data collection procedure, and the impact on measurements across contexts.

## Methods

### Study setting, design, and participants

This study is part of a multi-country project named SPICES—Scaling-up Packages of Interventions for Cardiovascular diseases in selected sites in Europe and sub-Saharan Africa—which aimed to implement a multi-component intervention in different contexts (https://www.uantwerpen.be/en/projects/spices/). The project is registered at clinicaltrails.gov (NCT03154736) and was conducted in selected areas of Belgium, England, France, South Africa, and Uganda. SPICES is an implementation research aiming to develop and implement packages of interventions through community engagement and primary healthcare facilities. Cardiovascular disease risk profiling and communication, coaching, health promotion, care and treatment, and self-management with follow-up are the core activities of the project. In this study, we particularly used data from participants’ CVD risk profiling of respective study areas before the intervention. Adults aged 18 to 75 years, non-pregnant, with no cognitive impairment were included in the study. Those who already experienced any CVD event were excluded from this analysis. Due to practical feasibility, we did not use strict proportionate sampling in each site and participants were selected on a voluntary basis. We used data from a total of 9309 participants, of which 4941 resided in Europe (Belgium: 350, England: 831, France: 3760), 3371 in South Africa, and 997 in Uganda. Details of the study area and methods have been published elsewhere.^18–23^

### Study approach

In Belgium, community settings and general practitioner practices in selected vulnerable districts of Antwerp were the entry points to reach participants for risk profiling. In England, organizations and venues, existing community groups, and workplaces in Brighton and Nottingham were used to reach participants and trained Community Health Volunteers (CHV) led the profiling activities. In France, the centre of the Brittany region of France—Brest—was selected, and workplaces, community events, and medico-social organizations were involved in participant recruitment. In South Africa, the Capricorn District of Limpopo Province was selected, and participants were reached through house-to-house visits in semi-urban and rural areas. In Uganda, two urban or semi-urban districts were selected, and profiling was performed using community health workers and primary care centres.

### Measurements

In order to measure participants’ risk of CVD, the NL-IHRS was employed,^13^ a validated score for quantifying risk factor burden and estimating future risk of CVD without the use of laboratory testing. The non-laboratory based tool has nine components: (1) age and sex, (2) smoking (active and passive), (3) DM, (4) HBP, (5) family history of CVD, (6) WHR, (7) psychosocial factors (stress, depression), (8) dietary factors, and (9) leisure time physical activity. Age, sex, and family history are non-modifiable, whereas the other risks are modifiable. Due to the relevance of lifestyle risk factors to the intervention as well as to clinicians and public health professionals, we preferred the NL-IHRS over other scores currently in use such as the Framingham risk score^24^ and the European SCORE.^25^ Moreover, this tool was chosen because of its simplicity to be used by non-medically trained people. The NL-IHRS considers dietary habits, physical activity, and psychosocial factors, which are the most important behavioural risk factors of CVD. The overall sum score of NL-IHRS ranges from 0 to 48, with higher scores indicating greater risk of experiencing CVD in the future. Details concerning the development and validation of the NL-IHRS are available in the original article^13^ and the tool is provided in the supplementary material (Supplementary material online, *Table S1*). Participants who scored <10 are considered to be at low risk, 10 to 15 at intermediate risk, and persons with a score of 16 or above are considered to be at high risk to develop CVD.

In all the sites except for Brighton, trained community healthcare workers, volunteers, or practice nurses collected the data. In Brighton, data were collected online by means of a self-completed tool. Waist and hip measurements were taken using standard procedures. History of DM and HBP was recorded based on self-reported assessments.

## Statistical analysis

Data were collected either in paper format or electronically using the Research Electronic Data Capture (REDCap) tool^26^ hosted at the University of Antwerp and Makerere University. The paper form questionnaire was also entered into REDCap and exported as a CSV file. Further data processing and analysis were performed using R statistical programming version 4.0.2.^27^ Categorical variables were summarized using absolute and relative frequencies. Numerical variables were checked for normality using Shapiro–Wilk’s normality test and graphically using a Q-Q plot. Then, we summarized these variables using either the mean and corresponding standard deviation (SD) in case of normality or using the median and interquartile range (IQR) in the absence thereof. Descriptive statistics were produced to describe the distribution of the main outcome, the NL-IHRS, and its implied risk categories (low, intermediate, and high).

Both the crude and adjusted mean NL-IHRS scores and percentage of participants in each risk category were compared across all sites. Considering the variations in participant characteristics among different contexts, we performed adjustments for age and sex. To calculate the adjusted mean score, we conducted regression analyses for individual component scores and the overall score, taking into account the region (Europe, South Africa, and Uganda) while adjusting for age and sex. Subsequently, we derived marginal means for each group based on the results of the regression analysis. Due to the similarity in healthcare systems and population characteristics, the results were pooled for three European countries (Belgium, France, and the UK), all of which belong to high-income nations. However, South Africa falls into the category of MICs, and Uganda is a LIC, resulting in distinct contextual differences, and were analysed separately. Furthermore, the variation in total and specific component scores (crude and adjusted) was compared across contexts using one-way analysis of variance (ANOVA). We categorized the INTERHEART components into four main categories: (1) non-modifiable (age, sex, and family history), (2) behavioural (smoking, second-hand smoke, dietary habit, physical activity), (3) metabolic and physical measures (DM, HBP, WHR), and (4) psychosocial (depression and stress). To determine the weight of each of these components, we computed the per cent contribution of each component of the score as compared to the total NL-IHRS score.

## Results

### Baseline characteristics of participants

A total of 9309 participants were involved in the current analysis. In *Table 1*, descriptive summary measures are provided with respect to age, sex, and risk classification based on the NL-IHRS for the European countries combined (Belgium, England, and France combined), South Africa, and Uganda. The median age of the participants was 56 years (IQR: 43–66), 49 years (IQR: 34–63), and 36 years (IQR: 27–49) in Europe (combining data from Belgium, England, and France), South Africa, and Uganda, respectively. Majorities were female, accounting for 62.3%, 74.6%, and 69.2% of all participants in Europe, South Africa, and Uganda, respectively. Approximately one-third (33.1%) and 16.4% of all participants in Europe were in the intermediate- or high-risk category, respectively. Whereas, 12.0% and 5.9% were classified in the high-risk category in South Africa and Uganda, respectively. Details of all country specific comparisons are available in the supplementary material (Supplementary material online, *Table S2*).

**Table 1:** Participants’ age, sex, and NL-IHRS risk category distribution in selected European and sub-Saharan African countries

Characteristic	Europe combined	South Africa	Uganda
Age, median (IQR)	56 (43–66)	49 (34–63)	36 (27–49)
Sex, *n* (%)			
Male	1756 (37.7)	839 (25.4)	306 (30.8)
Female	2905 (62.3)	2463 (74.6)	688 (69.2)
Risk category, *n* (%)			
Low (<10)	2277 (50.5)	1690 (51.1)	691 (69.5)
Intermediate (10 to 15)	1492 (33.1)	1220 (36.9)	244 (24.5)
High (≥16)	742 (16.4)	397 (12.0)	59 (5.9)

IQR, interquartile range.

### Variation in the total INTERHEART risk score and individual components

**Figure 1:**
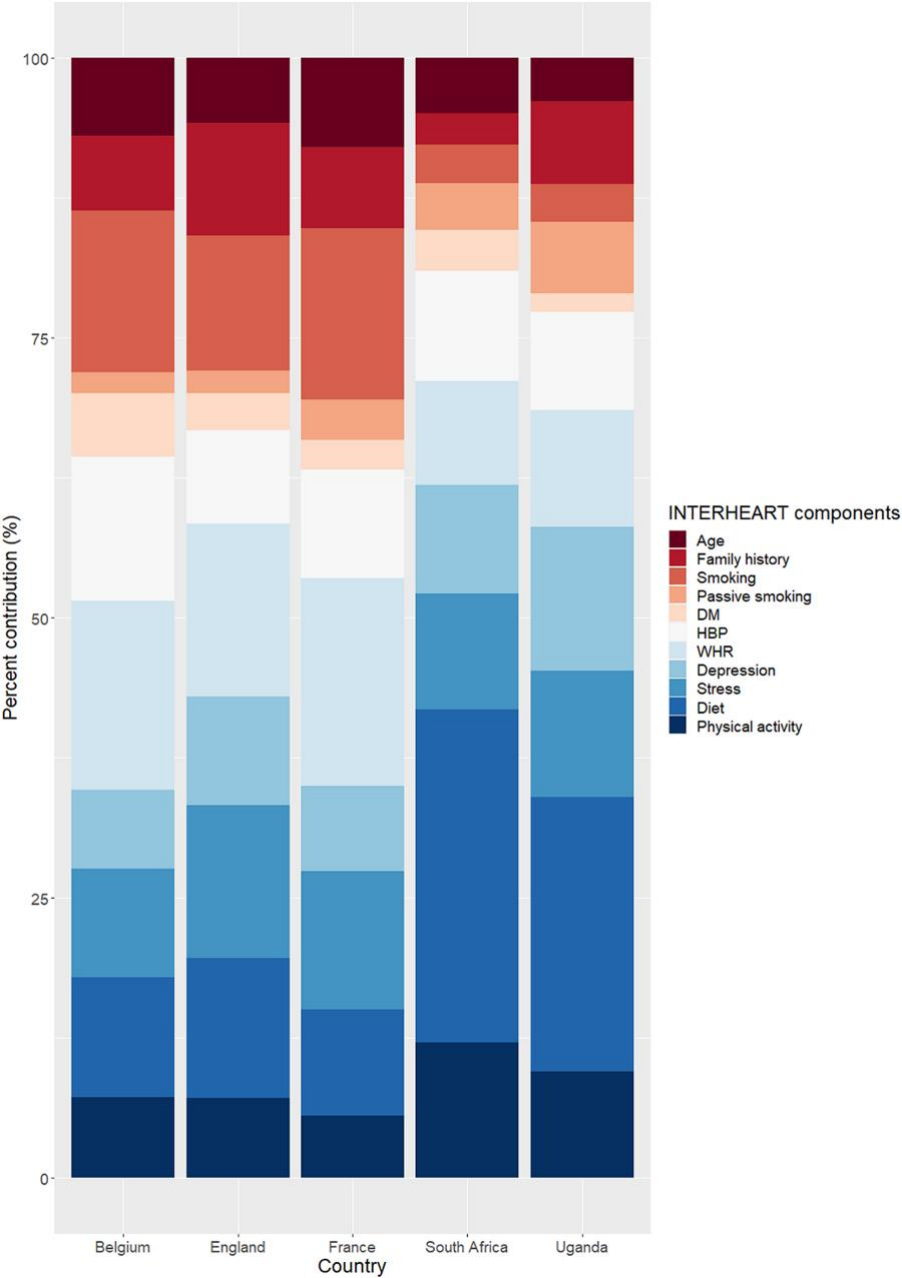
Percent contribution of each NL-IHRS component in selected European and sub-Saharan African countries. DM, diabetes mellitus; HBP, high blood pressure; NL-IHRS, Non-laboratory Interheart Risk Score; WHR, waist-to-hip ratio.

Details with regard to both crude and adjusted total and individual component scores for each context are summarized in *Table 2*. The mean NL-IHRS was found to be highest in Europe (10.2 points; 95%CI: 10.0, 10.4) and South Africa (10.2; 95%CI: 10.0, 10.3), but lowest in Uganda (7.6; 95%CI: 7.3, 7.9), with statistically significant difference (one-way ANOVA F-test *P* < 0.001). Regarding individual components, the score due to older age (≥65 years for females and ≥55 for males) was lowest in Uganda (mean: 0.29; 95%CI: 0.20, 0.38) followed by South Africa (0.54; 95%CI: 0.51, 0.57) and Europe (0.75; 95%CI: 0.73, 0.78). The score due to smoking was lower in sub-Saharan Africa, accounting for 0.26 (95%CI: 0.21, 0.31) in Uganda and 0.38 (0.37, 0.42) in South Africa, but highest in Europe (1.49; 95%CI: 1.42, 1.55), particularly in Belgium (1.82; 95%CI: 1.57, 2.07). Likewise, scores due to DM and HBP were lowest in Uganda, 0.13 (95%CI: 0.07, 0.18) and 0.66 (95%CI: 0.56, 0.77), respectively. Likewise, scores due to WHR was lowest in Uganda (0.79; 95%CI: 0.71, 0.86) and highest in Europe (1.81; 95%CI: 1.77, 1.86). Score related to dietary factors was much higher in South Africa (3.24; 95%CI: 3.19, 3.29) in comparison with Uganda (1.85; 95%CI: 1.78, 1.93) and Europe (1.04; 95%CI: 1.00, 1.07). Similarly, the average score due to low physical activity was highest in South Africa (1.32; 95%CI: 1.29, 1.35). Per cent contribution of subcomponents to the total score is summarized in *Figure 1*. Country specific comparisons are provided in the supplementary material (Supplementary material online, *Table S3*).

**Table 2:** Comparison of total and component mean scores of the NL-IHRS in selected European and sub-Saharan African countries

Measures	Europe		South Africa		Uganda		*P* value	*P* value^a^
	Crude	Adjusted	Crude	Adjusted	Crude	Adjusted		
Age	0.75 (0.73, 0.78)	—	0.54 (0.51, 0.57)	—	0.29 (0.20, 0.38)	—	<0.001	—
Family history	0.79 (0.74, 0.84)	0.77 (0.73, 0.82)	0.31 (0.26, 0.35)	0.32 (0.26, 0.38)	0.56 (0.47, 0.65)	0.62 (0.53, 0.72)	<0.001	<0.001
Smoking	1.49 (1.42, 1.55)	1.52 (1.47, 1.57)	0.38 (0.37, 0.42)	0.36 (0.30, 0.42)	0.26 (0.21, 0.31)	0.15 (0.04, 0.26)	<0.001	<0.001
Passive smoking	0.32 (0.30, 0.34)	0.34 (0.32, 0.36)	0.45 (0.42, 0.48)	0.44 (0.41, 0.47)	0.48 (0.43, 0.53)	0.40 (0.35, 0.45)	<0.001	0.134
Diabetes	0.31 (0.27, 0.35)	0.28 (0.24, 0.31)	0.40 (0.35, 0.46)	0.43 (0.38, 0.47)	0.13 (0.07, 0.18)	0.24 (0.16, 0.33)	<0.001	<0.001
HBP	0.99 (0.93, 1.05)	0.86 (0.81, 0.91)	1.07 (0.99, 1.16)	1.13 (1.05, 1.21)	0.66 (0.56, 0.77)	1.11 (0.99, 1.23)	<0.001	<0.001
WHR	1.81 (1.77, 1.86)	1.75 (1.71, 1.79)	1.01 (0.96, 1.06)	1.04 (0.99, 1.09)	0.79 (0.71, 0.86)	0.97 (0.88, 1.06)	<0.001	<0.001
Depression	0.81 (0.77, 0.84)	0.81 (0.77, 0.85)	1.06 (1.01, 1.10)	1.05 (1.00, 1.10)	0.97 (0.89, 1.06)	0.97 (0.88, 1.05)	<0.001	0.005
Stress	1.26 (1.22, 1.30)	1.26 (1.22, 1.30)	1.14 (1.08, 1.19)	1.14 (1.09, 1.19)	0.85 (0.77, 0.94)	0.82 (0.72, 0.91)	<0.001	<0.001
Dietary factors	1.04 (1.00, 1.07)	1.09 (1.05, 1.13)	3.24 (3.19, 3.29)	3.21 (3.17, 3.26)	1.85 (1.78, 1.93)	1.66 (1.57, 1.74)	<0.001	<0.001
Physical activity	0.62 (0.59, 0.64)	0.62 (0.59, 0.65)	1.32 (1.29, 1.35)	1.32 (1.28, 1.35)	0.72 (0.66, 0.78)	0.69 (0.63, 0.75)	<0.001	<0.001
Total score	10.2 (10.0, 10.4)	10.0 (9.8, 10.2)	10.2 (10.0, 10.3)	10.2 (10.0, 10.4)	7.6 (7.3, 7.9)	8.2 (7.8, 8.5)	<0.001	<0.001

Values are mean (95%CI).

HBP, high blood pressure; WHR, waist-to-hip ratio.

^a^Adjusted for age and sex.

To accommodate the age and sex distribution of the sample, we computed the total and component scores adjusted for age and sex. The adjusted mean NL-IHRS score was 10.0 (9.8, 10.2) in Europe and 10.2 (10.0, 10.4) in South Africa, which is higher than the score in Uganda (8.2 (7.8, 8.5)) (*P* < 0.001). The adjusted score due to smoking is higher in Europe (1.52, 95%CI: 1.47, 1.57) than South Africa (1.13, 95%CI: 1.05, 1.21) and Uganda (0.15, 95%CI: 0.04, 0.26) (*P* < 0.001). Likewise, score due to higher WHR was significantly higher in Europe (1.75; 95%CI: 1.71, 1.79) followed by South Africa (1.04; 95%CI: 0.99, 1.09) and Uganda (0.97; 95%CI: 0.88, 1.06). Score attributed to dietary factors was higher in South Africa (3.21; 95%CI: 3.17, 3.26) and Uganda (1.66; 95%CI: 1.57, 1.74), which is higher than Europe (1.09; 95%CI: 1.05, 1.13). Details of the adjusted component scores are available in *Table 2* below.

### Comparison of INTERHEART subcomponent categories and per cent contribution

**Figure 2:**
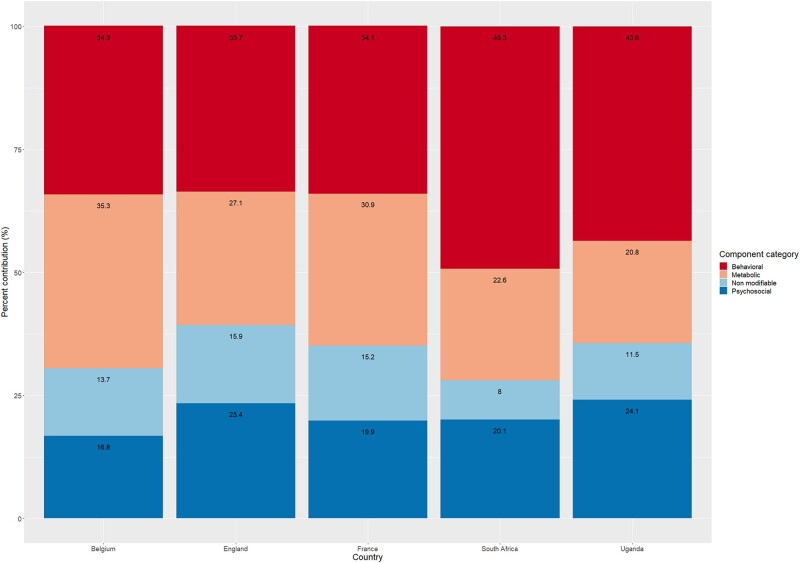
Percent contributions of each NL-IHRS category in selected European and sub-Saharan African countries.

Comparison of the total and subcomponent category scores for each context is summarized in *Table 3*. The NL-IHRS components were subcategorized into four (see above): non-modifiable, behavioural, metabolic and physical, and psychosocial. The average contribution coming from non-modifiable factors to the NL-IHRS was significantly higher in Europe (mean: 1.54; 95%CI: 1.49, 1.60; 15.2% of the total score) compared to Uganda (mean: 0.85, 95%CI: 0.72, 0.98; 11.5%) and South Africa (mean: 0.86; 95%CI: 0.80, 0.92; 8.0%). The per cent contribution of behavioural factors to the total score was higher in sub-Saharan African countries, both in South Africa (49.3%) and Uganda (43.6%), compared to Europe (34.0%). The per cent contribution of metabolic factors was lower in Uganda (20.8%) and South Africa (22.6%) compared to Europe (30.4%). The per cent contribution of psychosocial factors was higher in Uganda (24.1%), compared to South Africa (20.1%) and Europe (20.3), but the raw scores are almost similar. Contribution of components and categories was also assessed on different risk level (low, intermediate, and high) and the differences were consistent across risk groups. The per cent contributions of subcategories in each context are summarized in *Figure 2*. Details of country specific scores and per cent contributions are available in the supplementary material (Supplementary material online, *Table S4*).

**Table 3:** The NL-IHRS subcomponent categories and per cent contribution in selected European and sub-Saharan African countries

Measure	Maximum	Europe		South Africa		Uganda	
		Mean (95%CI)	%	Mean (95%CI)	%	Mean (95%CI)	%
Total score	48	10.2 (10.0, 10.4)	100.0	10.2 (10.0, 10.3)	100.0	7.6 (7.3, 7.9)	100.0
Non-modifiable	6	1.54 (1.49, 1.60)	15.2	0.86 (0.80, 0.92)	8.0	0.85 (0.72, 0.98)	11.5
Behavioural	21	3.46 (3.36, 3.55)	34.0	5.32 (5.23, 5.40)	49.3	3.31 (3.17, 3.44)	43.6
Metabolic	15	3.11 (3.02, 3.20)	30.6	2.46 (2.32, 2.60)	22.6	1.58 (1.43, 1.74)	20.8
Psychosocial	6	2.06 (2.00, 2.13)	20.3	2.18 (2.10, 2.25)	20.1	1.83 (1.70, 1.96)	24.1

All *P* values comparing Europe, South Africa and Uganda are <0.001.

## Discussion

In this study, we summarized the variation in total NL-IHRS and score due to each component in selected European and sub-Saharan African contexts representing low-, middle-, and high-income countries. We described the disparities in the contribution of component categories to the total risk score in respective contexts comparing the risk factor burden and identifying measurement variations. Overall, the total risk score is higher in HICs of Europe and middle-income country South Africa compared to low-income country Uganda. The NL-IHRS components greatly vary across contexts, in which behavioural factors contributed to a higher percentage of the total risk score in South Africa and Uganda. However, among behavioural factors, smoking (both active and passive) contributed the least in those two sub-Saharan African countries. Whereas, the contribution of an unhealthy diet and lack of leisure time physical activity is very high in sub-Saharan African countries particularly South Africa. This implies that, in those countries, lifestyle interventions could have a greater impact in primary prevention of CVDs. In contrast, the contribution of metabolic and physical factors is higher in European countries compared to sub-Saharan Africa.

The higher CVD risk burden in Europe and South Africa compared to Uganda could be due to the income per capita gradient across countries. Studies indicated that the burden of risk factors is highest in HICs, subsequently in middle- and low-income countries.^4,28^ In contrast, LMICs have a higher CVD incidence and mortality due to inadequate healthcare infrastructure.^4,7^ For instance, the use of cholesterol lowering agents and antihypertensive drugs for primary prevention of CVD is very low in LICs compared to HICs, leading to a higher rate of major CVD events and death.^4^ Although the burden of risk factors is higher in HICs, the rate of CVD events and mortality is relatively lower due to improved healthcare service and access to high-quality care. Thus, we suggest that the lower risk profile in LICs need to be supported by an integrated lifestyle and drug-based preventive services to minimize a large proportion of preventable deaths. In addition, employing direct measurement of blood pressure is recommended to minimize underestimation of the actual burden in resource-limited settings. Whereas in HICs, besides the clinical intervention, cost-effective public health programmes and promotion of healthy lifestyle could be beneficial to reduce premature mortality.

History of DM and HBP accounts for a higher weight of NL-IHRS, accounting for 5 and 6 out of 48, respectively. Per cent contribution of DM and HBP is very low in Uganda compared to South Africa and European countries, which could be attributed to the low prevalence of those risk factors. Participants in Uganda were generally of a younger age and the prevalence of DM and HBP is lower within these age groups, leading to lower scores attributed to these metabolic factors. Notably, in Uganda, the influence of age on the scores related to DM and HBP is evident, as the adjusted scores (0.24 and 1.11) were higher than the crude scores (0.13 and 0.66, respectively). On the other hand, the self-reporting nature of the tool might underestimate the real burden in LICs. Studies show that self-reported health assessments are inferior to objective measures particularly in LMICs.^29–31^ The majority of modifiable risk factors including hypertension, DM, and hyperlipidaemia remained underdiagnosed in low-income sub-Saharan African countries. Overall, above 40% of patients with diabetes and 60% of those with hypertension are not aware of their diagnosis before.^32,33^ Thus, the real prevalence of these metabolic risks and the overall NL-IHRS could be higher in LICs. Thus, triangulation of the self-reported data with direct measurements, particularly for HBP and DM, is recommended for more accurate risk stratification in LMICs.

Behavioural factors, particularly unhealthy dietary habits and physical inactivity during leisure time, took a large contribution to the NL-IHRS in sub-Saharan African countries, both South Africa and Uganda. Although the overall score was relatively lower in Uganda and South Africa, behavioural factors contribute nearly half of the total. Of behavioural factors, the burden and contribution of smoking are lower in sub-Saharan Africa compared to European countries. Spatial and temporal patterns of smoking also indicate that the burden of smoking is lower in sub-Saharan Africa (<5% in Uganda and 10% to 20% in South Africa) compared to Europe (20 to 40%).^34^ Besides, due to lower social acceptance of smoking in the region, the score related to smoking is subject to response bias which might result in under-reporting.

On the contrary, unhealthy dietary habits including frequent consumption of salty and/or deep-fried foods, inadequate fruit and vegetable consumption, and excessive meat/poultry consumption are highly prevalent and contribute to a large proportion of the total risk score in sub-Saharan Africa particularly South Africa. The Global Burden of Diseases (GBD) study also showed that dietary health risks such as low fruit and vegetable intake and high sodium intake are higher in sub-Saharan Africa compared with Northern and Central European countries.^35^ The exceptionally higher burden of dietary related risks in South Africa needs to be addressed through holistic interventions both at population and individual levels. Furthermore, the burden of physical inactivity and its contribution to the risk score is relatively higher in South Africa compared to European counterparts. Nearly two-thirds of participants reported that they are mainly sedentary or perform low levels of exercise during leisure time. A WHO global study using the standard physical activity measurement also put South Africa among the most inactive countries, with above 40% of the total population not fulfilling the recommended level.^36^

The per cent contribution of non-modifiable risks, i.e. age and family history of CVD, is lower in Uganda and South Africa compared to participants in Europe. This could be due to the relatively lower age of participants, lower prevalence of CVD risk factors, and/or under detection of risks in those countries. Self-reporting bias could also result in underestimation of age and family’s health status in LICs. Although, contribution of psychosocial factors including stress and depression to the total risk score is slightly higher in Uganda, the raw score is lowest. Thus, the higher per cent contribution is mainly due to a lower total score in Uganda compared to other countries.

### Methodological and tool considerations

Overall, the NL-IHRS tool is easy to use and provides vital information on individuals’ future risk of CVD. The tool is validated and has acceptable performance in comparison with the fasting cholesterol-based INTERHEART risk score (FC-IHRS) with regional calibration.^37^ Besides estimating the risk level, the specific items are a useful tool during preventive intervention to improve risk awareness and to educate and coach intermediate- and high-risk groups targeting those specific risk factors. In particular, this tool would have a tremendous advantage in resource-limited settings to screen participants’ levels of CVD risk without any laboratory procedure. Nevertheless, this study identified substantial variations in the total NL-IHRS as well as its subcomponents in European and sub-Saharan African countries, implying that specific contexts need to be taken into consideration in utilizing the tool. Part of these differences could be explained by the variation in the real prevalence, while the measurement technique might also be the contributing factor.

Except for waist and hip measurements, most of the NL-IHRS tool components are self-reported. Historical recall of some components such as diabetes, hypertension, smoking, and depression may be inferior to direct measurements, particularly in low-income settings due to lack of regular medical check-up, low literacy level, and social desirability biases. Studies indicate that self-reported measures underestimate the prevalence of clinical outcomes in LICs and low socioeconomic groups of middle- and high-income countries.^38,39^ Participants’ level of education, age group, sex, previous health condition, and family history are among the factors that influence the accuracy of self-report measures.^31,39^ Thus, using both self-reported and direct measurements (e.g. blood glucose and blood pressure) could improve the accuracy in low-income settings. Furthermore, the physical activity component of NL-IHRS only asks about leisure time activity, i.e. other work and transport related activities are not considered. This might lead to underestimation of the actual physical activity level particularly in low-income settings of Uganda and South Africa as most of them are involved in physically demanding labour activities. Therefore, minor modification of specific items might be needed to customize the tool in different contexts.

The findings from this study should be interpreted considering the following limitations. First, the data sources were not representative for respective countries and results should not be considered as a population estimate for that context. Nevertheless, this study investigates differences in both total and component risk scores across various contexts, offering insights for the practical application of risk assessment tools. Second, participant recruitment and sampling approaches vary across settings which might result in differences in total risk scores and also component scores, since some recruitment strategies led to more high-risk participants than others. Third, in most of the settings, females are over-represented in the samples which might underestimate the population level risk score, as the age cut-off varies according to sex, 65 for females and 55 for males. Fourth, the NL-IHRS tool was translated into different languages according to the context, which might result in variation in responses. Finally, for comparison purposes, we summarized results for Europe, South Africa, and Uganda separately. Thus, the estimates for Europe only refer to those three Western European countries, i.e. the findings are not representative of other European countries or regions and the total and component scores might be different particularly in Eastern European countries.

## Conclusions

In general, the total risk score, components, categories, and their contribution to the total score greatly vary in European and sub-Saharan African countries. The total risk score is higher in Europe and South Africa compared to Uganda. Behavioural factors contribute largely to the total risk score in sub-Saharan African countries, particularly the burden of dietary factors and physical inactivity are higher in South Africa. Thus, the causes of such a high burden of risk factors need to be investigated thoroughly and scaling up of effective intervention strategies is recommended. The burden of smoking is higher in Europe, and its contribution to the total score is higher than in sub-Saharan African countries. The per cent contribution of DM and HBP is higher in Europe followed by South Africa and Uganda, which could be due to variation in risk factor burden or the self-reporting bias in resource-limited settings. Therefore, primary preventive initiatives should identify the burden of risk factors across contexts and intervention activities need to be customized accordingly to optimize uptake and effectiveness. Moreover, contextualizing the risk assessment tool and evaluating its usefulness in different settings are recommended. The measurement techniques of each NL-IHRS component need to be adapted to fit the context both in LMICs and HICs. Triangulating the self-report components such as DM and HBP with direct measurement might improve the estimation in those settings.

## Supplementary Material

oead131_Supplementary_Data

## Data Availability

The datasets used and/or analysed during the current study are available from the corresponding author on reasonable request.

## Lead author biography

Hamid Y. Hassen is a dedicated researcher with a keen interest in the epidemiology of chronic non-communicable diseases. His current research is centred on investigating the risk factors associated with non-communicable diseases, particularly focusing on cardiovascular diseases and cancer. He is actively involved in the development and validation of prediction models, as well as the implementation of prevention strategies. Hamid also dedicates his efforts to advancing epidemiological and statistical methods and evaluating tools for measuring health outcomes. Currently, Hamid holds a research position at the University of Antwerp, where he earned both his Master of Epidemiology and PhD. He is known for his frequent collaborations with fellow researchers who specialize in epidemiological methods, non-communicable disease prevention, and the study of disparities in cancer survival across different settings.

## Appendix

**SPICES consortium collaborators**

University of Antwerp (Hilde Bastiaens, Hamid Y. Hassen, Naomi Aerts, Sibyl Anthierens, Kathleen Van Royen, Caroline Masquillier); Brest University (Jean Yves Le Reste, Delphine Le Goff, Gabriel Perraud); University of Sussex (Harm van Marwijk, Elisabeth Ford, Tom Grice-Jackson, Imogen Rogers, Papreen Nahar); Nottingham Trent University (Linda Gibson, Mark Bowyer, Almighty Nkengateh); Makerere University (Geofrey Musinguzi, Rawlance Ndejjo, Fred Nuwaha); University of Limpopo (Tholene Sodi, Peter M. Mphekgwana, Nancy Malema, Nancy Kgatla, Tebogo M. Mothiba).
