# Supplementary material for: Disparities in the non-laboratory INTERHEART risk score and its components in selected countries of Europe and sub-Saharan Africa: analysis from the SPICES multi-country project
Source: Eur Heart J Open. 2023 Dec 5;3(6):oead131. doi: 10.1093/ehjopen/oead131 (PMC10733186; doi:10.1093/ehjopen/oead131)
Supplement: oead131_Supplementary_Data [file oead131_supplementary_data.pdf]

Supplementary material: Disparities in the non-laboratory INTERHEART risk score and its components in selected countries of Europe and sub-Saharan Africa: Analysis from the SPICES multi-country project

Hamid Y. Hassen[a*], Steven Abrams[a,b¥], Geofrey Musinguzi[a,d], Imogen Rogers[c], Alfred Dusabimana[a], Peter M. Mphekgwana[e], and Hilde Bastiaens[a¥] on behalf of the Scaling-up Packages of Interventions for Cardiovascular diseases in Europe and Sub-Saharan Africa (SPICES) study investigators[¶]

[a] Department of Family Medicine and Population Health, Faculty of Medicine and Health Sciences, University of Antwerp, Antwerp, 2610, Belgium

[b] Interuniversity Institute for Biostatistics and statistical Bioinformatics, Data Science Institute, Hasselt University, Diepenbeek, 3590, Belgium

[c] Department of Primary Care and Public Health, Brighton and Sussex Medical School, Brighton, United Kingdom

[d] Department of Disease Control and Environmental Health, School of Public Health, Makerere University, Kampala, Uganda

[e] Research Administration and Development, University of Limpopo, Polokwane 0700, South Africa

[¶] Investigators listed at the end of the paper

***Correspondence to**: Hamid Y. Hassen (Hamid.hassen@uantwerpen.be)

Department of Family Medicine and Population Health, University of Antwerp, Antwerp, 2610, Belgium

¥ - These authors share senior co-authorship

[¶ -] Investigators listed at the end of the paper

Contents

Contents 2
Measurement tool.. 3
Extended results.. 5

Measurement tool

Table S1. The “Non laboratory” based Modifiable Risk score (Source: https://doi.org/10.1093/eurheartj/ehq448)

Risk factor	Question	Points for the answer		Points for each section
**Age**	Are you a man 55 years or older OR woman 65 years or older?		2	Points
	OR Are you a man younger than 55 years or woman younger than 65 years		0	
**Smoking. Pick the description which matches you best:**	I never smoked		0	Points
	OR I am a former smoker (last smoked more than 12 months ago)		2	
	OR I am a current smoker or I smoked regularly in the last 12 months, and I smoke…	1-5 cigarettes per day	2	
		6-10 cigarettes per day	4	
		11-15 cigarettes per day	6	
		16-20 cigarettes per day	7	
		More than 20 cigarettes per day	11	
**Second hand smoke**	Over the past 12 months, what has been your typical exposure to other people’s tobacco smoke?	Less than 1 hour or exposure per week or no exposure	0	Points
		OR One or more hours of secondhand smoke exposure per week	2	
**Diabetes**	Do you have diabetes mellitus?	Yes	6	Points
		No or unsure	0	
**High Blood Pressure**	Do you have high blood pressure	Yes	5	Points
		No or unsure	0	
**Family history**	Have either or both of your biological parents had a heart attack?*	Yes	4	Points
		No or unsure	0	
**Waist to hip ratio**	Pick one only:	Quartile 1: Less than 0.873	0	Points
		Quartile 2 &3: 0.873 - 0.963	2	
		Quartile 4: greater than or =0.964	4	
**Psychosocial factors**	How often have you felt work or home life stress in the last year? Pick one only	Never or some periods	0	Points
		OR Several periods of stress or permanent stress	3	
	During the past 12 months, was there ever a time when you felt sad, blue, or depressed for two weeks or more in a row?	Yes	3	Points
		No	0	

**Dietary factors. Pick one answer for each food group mentioned**	Do you eat salty food or snacks one or more times a day	Yes	1	Points
		No	0	
	Do you eat deep fried foods or snacks or fast foods 3 or more times a week?	Yes	1	Points
		No	0	
	Do you eat fruit one or more times daily?	Yes	0	Points
		No	1	
	Do you eat vegetables one or more times daily?	Yes	0	Points
		No	1	
	Do you eat meat and/ or poultry 2 or more times daily?	Yes	2	Points
		No	0	
**Physical activity**	How active are you during your leisure time?	I am mainly sedentary or perform mild exercise (requiring minimal effort)	2	Points
		OR I perform moderate or strenuous physical activity in my leisure time	0	

Extended results

Table S2. Participants' age, sex and NL-IHRS risk category distribution in selected European and sub-Saharan African Countries.

Characteristic	Belgium	England	France	Europe combined	South Africa	Uganda
Age (median ± IQR)	57 (16)	54 (24)	56 (24)	56 (23)	49 (29)	36 (22)
Sex, n(%)						
Male	161 (46.7)	289 (31.1)	1306 (37.4)	1756 (37.7)	839 (25.4)	306 (30.8)
Female	184 (53.3)	530 (68.9)	2191 (62.6)	2905 (62.3)	2463 (74.6)	688 (69.2)
Risk category, n(%)						
Low (<10)	120 (34.6)	340 (43.5)	1817 (53.7)	2277 (50.5)	1690 (51.1)	691 (69.5)
Intermediate (10 to 15)	126 (36.3)	289 (37.0)	1077 (31.8)	1492 (33.1)	1220 (36.9)	244 (24.5)
High (≥16)	101 (29.1)	152 (19.5)	489 (14.4)	742 (16.4)	397 (12.0)	59 (5.9)

IQR: Interquartile Range

Table S3. Comparison of component scores of the NL-INTERHEART risk score in selected European and sub-Saharan African Countries.

**Measures**	**Europe combined**	**Belgium**	**England**	**France**	**South Africa**	**Uganda**
Age	0.75 (0.73, 0.78)	0.88 (0.78, 0.98)	0.63 (0.57, 0.70)	0.77 (0.74, 0.80)	0.54 (0.51, 0.57)	0.29 (0.20, 0.38)
Family history	0.79 (0.74, 0.84)	0.85 (0.67, 1.02)	1.12 (0.99, 1.24)	0.71 (0.65, 0.76)	0.31 (0.26, 0.35)	0.56 (0.47, 0.65)
Smoking	1.49 (1.42, 1.55)	1.82 (1.57, 2.07)	1.33 (1.21, 1.45)	1.49 (1.42, 1.56)	0.38 (0.37, 0.42)	0.26 (0.21, 0.31)
Passive smoking	0.32 (0.30, 0.34)	0.23 (0.17, 0.30)	0.22 (0.18, 0.27)	0.35 (0.33, 0.38)	0.45 (0.42, 0.48)	0.48 (0.43, 0.53)
Diabetes	0.31 (0.27, 0.35)	0.72 (0.51, 0.92)	0.37 (0.27, 0.47)	0.25 (0.21, 0.29)	0.40 (0.35, 0.46)	0.13 (0.07, 0.18)
Hypertension	0.99 (0.93, 1.05)	1.63 (1.38, 1.87)	0.92 (0.78, 1.05)	0.94 (0.88, 1.01)	1.07 (0.99, 1.16)	0.66 (0.56, 0.77)
WHR	1.81 (1.77, 1.86)	2.13 (1.98, 2.28)	1.71 (1.61, 1.82)	1.81 (1.76, 1.86)	1.01 (0.96, 1.06)	0.79 (0.71, 0.86)
Depression	0.81 (0.77, 0.84)	0.89 (0.75, 1.04)	1.07 (0.97, 1.17)	0.74 (0.69, 0.78)	1.06 (1.01, 1.10)	0.97 (0.89, 1.06)
Stress	1.26 (1.22, 1.30)	1.23 (1.07, 1.38)	1.51 (1.41, 1.62)	1.20 (1.15, 1.25)	1.14 (1.08, 1.19)	0.85 (0.77, 0.94)
Diet	1.04 (1.00, 1.07)	1.36 (1.21, 1.51)	1.38 (1.28, 1.47)	0.92 (0.88, 0.96)	3.24 (3.19, 3.29)	1.85 (1.78, 1.93)
Exercise	0.62 (0.59, 0.64)	0.91 (0.80, 1.01)	0.79 (0.72, 0.86)	0.54 (0.51, 0.57)	1.32 (1.29, 1.35)	0.72 (0.66, 0.78)
Total score	10.2 (10.0, 10.4)	12.5 (11.9, 13.2)	11.0 (10.6, 11.4)	9.7 (9.5, 9.9)	10.2 (10.0, 10.3)	7.6 (7.3, 7.9)

Values are mean (95%CI); P value comparing Europe, SA and Uganda are all <0.001

Table S3. Comparison of adjusted component scores of the NL-INTERHEART risk score in selected European and sub-Saharan African Countries. (adjusted for age)

Measures	Europe combined	Belgium	England	France	South Africa	Uganda	P value[a]	P value[b]
Family history	0.77 (0.73, 0.82)	0.82 (0.66, 0.97)	1.10 (1.01, 1.20)	0.69 (0.64, 0.74)	0.32 (0.26, 0.38)	0.62 (0.53, 0.72)	<0.001	0.236
Smoking	1.52 (1.47, 1.57)	1.87 (1.69, 2.05)	1.35 (1.24, 1.47)	1.52 (1.46, 1.58)	0.36 (0.30, 0.42)	0.15 (0.04, 0.26)	<0.001	0.013
Passive smoking	0.34 (0.32, 0.36)	0.28 (0.20, 0.36)	0.24 (0.19, 0.29)	0.37 (0.35, 0.40)	0.44 (0.41, 0.47)	0.40 (0.35, 0.45)	0.134	0.084
Diabetes	0.28 (0.24, 0.31)	0.64 (0.50, 0.78)	0.35 (0.26, 0.44)	0.22 (0.17, 0.27)	0.43 (0.38, 0.47)	0.24 (0.16, 0.33)	<0.001	0.031
Hypertension	0.86 (0.81, 0.91)	1.35 (1.16, 1.55)	0.84 (0.71, 0.96)	0.82 (0.75, 0.88)	1.13 (1.05, 1.21)	1.11 (0.99, 1.23)	<0.001	0.012
WHR	1.75 (1.71, 1.79)	2.01 (1.86, 2.15)	1.67 (1.58, 1.77)	1.75 (1.70, 1.79)	1.04 (0.99, 1.09)	0.97 (0.88, 1.06)	<0.001	<0.001
Depression	0.81 (0.77, 0.85)	0.90 (0.75, 1.04)	1.08 (0.98, 1.17)	0.74 (0.69, 0.78)	1.05 (1.00, 1.10)	0.97 (0.88, 1.05)	0.005	0.097
Stress	1.26 (1.22, 1.30)	1.24 (1.08, 1.39)	1.52 (1.42, 1.62)	1.20 (1.15, 1.25)	1.14 (1.09, 1.19)	0.82 (0.72, 0.91)	<0.001	<0.001
Diet	1.09 (1.05, 1.13)	1.47 (1.34, 1.60)	1.42 (1.33, 1.50)	0.98 (0.93, 1.02)	3.21 (3.17, 3.26)	1.66 (1.57, 1.74)	<0.001	<0.001
Exercise	0.62 (0.59, 0.65)	0.92 (0.82, 1.02)	0.80 (0.73, 0.86)	0.55 (0.52, 0.58)	1.32 (1.28, 1.35)	0.69 (0.63, 0.75)	<0.001	<0.001
Total score	10.0 (9.8, 10.2)	12.1 (11.5, 12.6)	10.8 (10.5, 11.2)	9.5 (9.3, 9.7)	10.2 (10.0, 10.4)	8.2 (7.8, 8.5)	<0.001	<0.001

Values are adjusted mean (95%CI); P value comparing Europe, SA and Uganda are all <0.001
Adjusted for age and sex

a- p value between Europe, SA and Uganda; b- p value between countries

Table S4. Comparison of subcomponent category scores and percent contribution of the NL-INTERHEART risk score in selected European and sub-Saharan African Countries.

**Measure**	**Belgium**		**England**		**France**		**Europe combined**		**South Africa**		**Uganda**	
	M(95%CI)	%	M(95%CI)	%	M(95%CI)	%	M(95%CI)	%	M(95%CI)	%	M(95%CI)	%
Total score	12.5 (11.9, 13.2)		11.0 (10.6, 11.4)		9.7 (9.5, 9.9)		10.2 (10.0, 10.4)		10.2 (10.0, 10.3)		7.6 (7.3, 7.9)	
Non-modifiable	1.73 (1.52, 1.93)	13.7	1.76 (1.61, 1.90)	15.9	1.48 (1.41, 1.54)	15.2	1.54 (1.49, 1.60)	15.2	0.86 (0.80, 0.92)	8.0	0.85 (0.72, 0.98)	11.5
Behavioural	4.32 (3.94, 4.71)	34.3	3.72 (3.51, 3.92)	33.7	3.31 (3.19, 3.42)	34.1	3.46 (3.36, 3.55)	34.0	5.32 (5.23, 5.40)	49.3	3.31 (3.17, 3.44)	43.6
Metabolic	4.46 (4.06, 4.85)	35.3	2.99 (2.76, 3.22)	27.1	3.00 (2.90, 3.10)	30.9	3.11 (3.02, 3.20)	30.6	2.46 (2.32, 2.60)	22.6	1.58 (1.43, 1.74)	20.8
Psychosocial	2.12 (1.86, 2.37)	16.8	2.58 (2.41, 2.75)	23.4	1.94 (1.86, 2.01)	19.9	2.06 (2.00, 2.13)	20.3	2.18 (2.10, 2.25)	20.1	1.83 (1.70, 1.96)	24.1

Non-modifiable: Age, family history
Behavioural: Dietary factors, smoking, physical activity
Metabolic: Diabetes mellitus, high blood pressure, waist-hip-ratio,
Psychosocial: depression, stress
